# SensRORing cholesterol to drive protumoral myelopoiesis

**DOI:** 10.1002/1878-0261.70113

**Published:** 2025-08-20

**Authors:** Sara Gennari, Luigi Nezi, Teresa Manzo

**Affiliations:** ^1^ Department of Molecular Biotechnology and Health Sciences University of Turin Italy; ^2^ Department of Experimental Oncology Istituto Europeo di Oncologia IRCCS Milano Italy

**Keywords:** anti‐tumor response, lipid metabolism, Protumoral myelopoiesis, RORγ

## Abstract

Protumoral myelopoiesis is a determinant of immunoevasion and tumor spread in many malignancies. In a recent issue of *Cancer Discovery*, Bleve and colleagues point to cholesterol‐driven RORγ activation as the molecular trigger of myeloid‐derived suppressor cells (MDSCs) and tumor‐associated macrophages (TAMs) expansion, resulting in defective antitumor response and disease progression.

## Graphical abstract

Bleve et al. uncover a cholesterol‐driven immune evasion pathway, where RORγ‐dependent myelopoiesis shapes tumor immunosuppression. Targeting RORγ and PCSK9 emerges as a strategy to boost cancer immunotherapy.

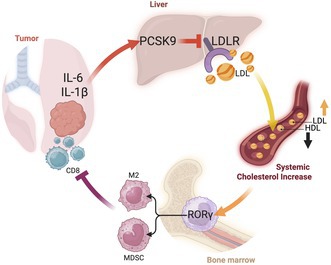

**Abbreviations**
ACAT1: acetyl‐CoA acetyltransferase 1
CCL2: C‐C motif chemokine ligand 2
CCR2: C‐C motif chemokine receptor 2
CSF1: colony‐stimulating factor 1
CSF1R: colony‐stimulating factor 1 receptor
CTLs: cytotoxic T lymphocytes
HMG‐CoA: 3‐hydroxy‐3‐methylglutaryl‐coenzyme A
IL‐1β: interleukin 1 beta
IL‐6: interleukin 6
LDL: low‐density lipoprotein
LXR: liver X receptor
MDSCs: myeloid‐derived suppressor cells
MHC‐I: major histocompatibility complex class I
M‐MDSCs: monocytic myeloid‐derived suppressor cells
NLR: neutrophil‐to‐lymphocyte ratio
PCSK9: proprotein convertase subtilisin/kexin type 9
PD‐1: programmed cell death protein 1
PD‐L1: programmed cell death‐ligand 1
RORC1: RAR‐related orphan receptor C isoform 1
RORγ: retinoic acid–related orphan receptor gamma
TAMs: tumor‐associated macrophages
Th17: T helper 17 (cells)
TME: tumor microenvironment

The relationship between cancer progression and myelopoiesis is immunologically driven, and tumor‐induced systemic accumulation and polarization of myeloid cells toward an immunosuppressive phenotype is a potent driver of metastasis formation [1, 2, 3]. Clinically, systemic accumulation of myeloid cells is strongly correlated with poor prognosis in multiple solid malignancies, and a high neutrophil‐to‐lymphocyte ratio (NLR) is consistently associated with adverse outcomes [4, 5]. Thus, abnormal myelopoiesis supports tumor growth and spread; however, the molecular mechanisms underlying this expansion remain poorly understood.

A deregulated lipid metabolism is a common feature of cancer cells [6] and, as a double‐edged sword, it greatly influences immune cells [7]. For instance, perturbation of cholesterol metabolism via LXR agonist [8], ACAT1 [9], or HMG‐CoA reductase inhibition by statins [10] empowers cytotoxic T lymphocytes (CTLs) with a superior anticancer response in several tumor models and, when combined with anti‐PD1 therapy, improves tumor control and long‐lasting survival. Indeed, differently from monotherapies, these combinations act simultaneously on the TME and on intratumoral CTLs, suggesting that mastering metabolic cues that imprint an abnormal myelopoiesis has the potential to re‐establish intratumoral CTLs functional fitness. Previous studies from this research group provided insight into regulation of pathological myelopoiesis by one of the members of the nuclear receptor superfamily and already suggested that RORC1 could be a key driver of MDSCs and TAMs differentiation [2]. Now, Bleve et al. [11] unveil a mechanistic axis by which tumor, lipid metabolism, and immune suppression converge via the cholesterol‐sensitive transcription factor RORγ.

The study indicates the nuclear receptor retinoic acid–related orphan receptor gamma (RORγ) as a central metabolic‐immune sensor, activated by cholesterol in response to inflammatory signaling from tumors. This activation drives the expansion of myeloid‐derived suppressor cells (MDSCs) and tumor‐associated macrophages (TAMs), establishing a tolerogenic tumor microenvironment.

In particular, inflammatory tumor‐derived IL‐1β and IL‐6 promote hepatic expression of PCSK9, which degrades LDL receptors and elevates systemic cholesterol levels. The subsequent hypercholesterolemia activates RORγ in myeloid progenitors, skewing their differentiation toward immunosuppressive cells. The result is a tumor‐favorable immunologic environment marked by suppressed T‐cell activity, enhanced metastatic potential, and resistance to checkpoint blockade.

Through the rigorous integration of transplantable and genetically engineered murine cancer models, along with dietary and pharmacologic modulation, the authors provide a detailed map of the tumor–liver–bone marrow axis. Notably, the use of bone marrow chimeras to distinguish the hematopoietic from the hepatic contribution establishes a robust causal framework and, combined with luciferase reporter assays and cytokine neutralization strategies, it positions RORγ as a central metabolic‐immune sensor linking systemic cholesterol dysregulation and immunosuppressive myelopoiesis to tumor‐induced inflammation. The mechanistic evidence produced in this study may also contribute to the longstanding paradox of obese cancer patients having better survival outcomes during treatment, especially those treated with immune checkpoint blockade [12].

On the other hand, the reversal of myeloid‐driven immunosuppression and restoration of CD8^+^ T cell activity following RORγ inhibition with its antagonist SR2211, as well as PCSK9 blockade, lays the foundation for future translational studies, especially considering the preliminary evidence that therapeutic targeting of RORγ may also enhance the efficacy of anti‐PD‐1 blockade in mice. The clinical relevance of these results is further strengthened by the correlation between elevated levels of RORγ^+^ M‐MDSCs, PCSK9, and levels of serum cholesterol in patients with non‐small‐cell lung cancer. However, these data should be confirmed on larger cohorts, and whether RORγ inhibition can directly reverse immunosuppressive phenotypes on patient‐derived cells has to be established. For instance, it would be important to assess whether targeting PCSK9 or RORγ could rescue MHC‐I downregulation or upregulate PD‐L1 expression on patient‐derived tumoroids, respectively.

While the RORγ axis is convincingly positioned as a central mediator of lipid‐induced immunosuppression, its interaction with other well‐characterized pathways (i.e., CSF1/CSF1R and CCL2/CCR2) and with different myeloid populations that may undergo functional rather than quantitative changes (i.e., neutrophils) warrant further exploration and could potentially provide a more integrated therapeutic perspective. In addition, RORγ plays essential roles in T‐cell biology, especially in the differentiation and function of Th17 cells. Although the myeloid‐specific knockout clarifies compartmental contributions, systemic administration of SR2211 may affect lymphoid compartments. Thus, the broader immunological consequences of RORγ inhibition remain unexplored. Similarly, while the authors suggest that statins may be ineffective due to their distinct mechanism (targeting HMG‐CoA reductase rather than PCSK9), this hypothesis has not been experimentally tested yet. A direct comparison between statins and PCSK9‐targeting strategies would strengthen the rationale for prioritizing PCSK9 as a therapeutic target.

In summary, by identifying RORγ as a cholesterol‐sensitive driver of immunosuppressive myelopoiesis, Bleve et al. expand the conceptual landscape of immunometabolism and identify RORγ as a promising therapeutic target at the interface of lipid metabolism and immune regulation (Fig. 1). The elegant and detailed mechanistic analysis advances our understanding of how tumors exploit systemic metabolic pathways to evade immune surveillance. With continued translational and clinical investigation, this work may pave the way for novel combination therapies that reprogram the immune microenvironment in metabolically dysregulated cancers.

**Fig. 1:**
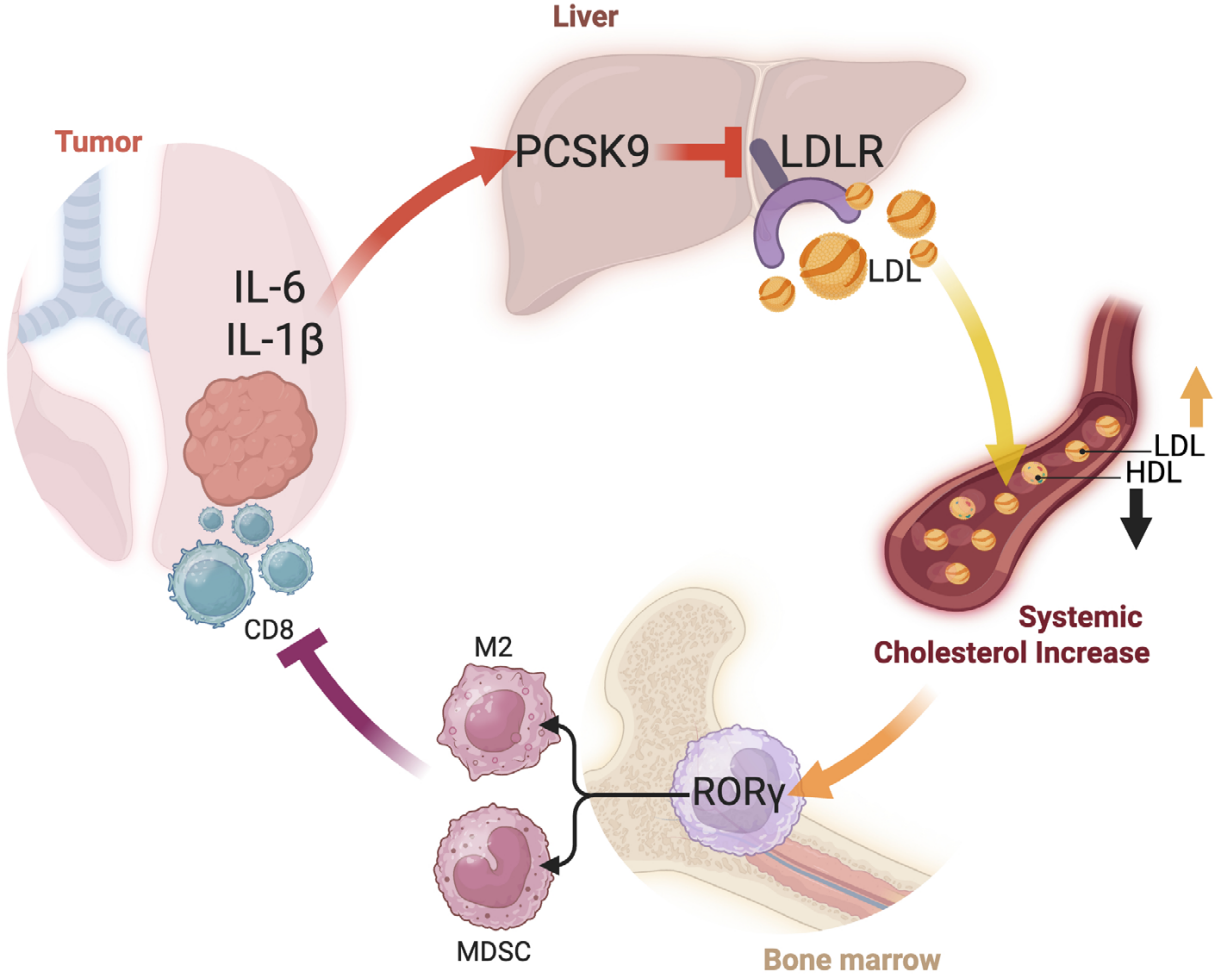
Cholesterol‐driven RORγ activation restrains the anti‐tumor potential of CD8^+^ T cells. Cholesterol‐induced RORγ activation is a key driver of protumoral myelopoiesis, promoting MDSC and TAM expansion and thereby facilitating immune evasion and tumor progression. Myeloid‐derived suppressor cells (MDSCs); tumor associated macrophages (TAM).

## Conflict of interest

The authors declare no conflict of interest.

## Author contributions

SG, LN, and TM have made a substantial, direct, and intellectual contribution to the work; and approved it for publication.
